# Navigating Challenges in Adult Airway Foreign Body Extraction: Retrospective Insights From Flexible Bronchoscopy

**DOI:** 10.7759/cureus.76173

**Published:** 2024-12-22

**Authors:** Ram N Jalandra, Kunal Deokar, Nishant Kumar Chauhan, Naveen Dutt

**Affiliations:** 1 Department of Pulmonary Medicine, All India Institute of Medical Sciences, Bathinda, Bathinda, IND; 2 Department of Pulmonary Medicine, All India Institute of Medical Sciences, Rajkot, Rajkot, IND; 3 Department of Pulmonary Medicine, All India Institute of Medical Sciences, Jodhpur, Jodhpur, IND

**Keywords:** airway, bronchoscopy, challenges, extraction, foreign body

## Abstract

Background

Airway foreign body aspiration is an emergency predominantly observed in children and the elderly. However, it also occurs in adults, presenting with a variety of symptoms. Both rigid and flexible bronchoscopies are employed for foreign body retrieval. In this context, we share our experience with foreign body extraction using flexible bronchoscopy in adult patients.

Methods

We conducted a retrospective study from January 2018 to August 2019 in the bronchoscopy suite of our institute's Department of Pulmonary Medicine. This study centered on the total bronchoscopies performed, the cases where foreign bodies were visualized, the location of these bodies, the techniques used for their retrieval, and the associated complications.

Results

In this retrospective analysis of 510 bronchoscopic procedures, 7.6% (n = 39) were conducted for the suspicion of foreign body aspiration. Foreign bodies were detected in 2.1% (n = 11) of these cases. The mean age of the patients was 44.27 ± 17.3 years, with 63.6% (n = 7) being male. Organically derived materials constituted the majority of the retrieved foreign bodies, accounting for 91% (n = 10) of cases. Flexible bronchoscopy facilitated the successful extraction of foreign bodies in 72.7% (n = 8) of these instances, albeit with some complications noted during the procedures.

Conclusions

Diagnosis and management of airway foreign body aspiration in adults demand keen vigilance. Flexible bronchoscopy has showcased its effectiveness in foreign body extraction, highlighting its role as a primary intervention tool. Typically, a combination of extraction tools is utilized to remove airway foreign bodies.

## Introduction

Accidental impaction of organic or inorganic material into airways results in airway foreign bodies. Such incidents are a common emergency and pose significant challenges for both diagnosis and management. Foreign body aspiration is particularly frequent in vulnerable populations such as children and the elderly, mainly due to their inability to protect their airways effectively [1]. In adults, the presentation of such cases can be quite variable. They may manifest overtly with symptoms such as acute onset of cough, breathlessness, and wheezing. On the other hand, some may exhibit more insidious symptoms, or in certain instances, the foreign body might be discovered incidentally during investigations for unrelated conditions. 

Given the potential variability in presentation, it becomes important for clinicians to maintain a high degree of suspicion, especially in adults. Anecdotal accounts or patient history indicating events suggestive of foreign body aspiration, like choking episodes while eating, can offer valuable diagnostic clues. However, the absence of clear symptoms can lead to missed or delayed diagnoses, resulting in complications such as non-resolving pneumonia, recurrent chest infections, lung abscesses, or even focal bronchiectasis. Such outcomes highlight the importance of prompt and accurate identification of these cases. 

The nature of the foreign body (its size, shape, and composition), its location within the airway, patient-related factors (such as underlying respiratory conditions and anesthesia risk), and the availability of specialized equipment (such as cryo-probes) all contribute to the complexity of management. Moreover, variations in operator skill level, institutional protocols, and the need for multidisciplinary collaboration (involving pulmonologists, anesthesiologists, and sometimes otolaryngologists or thoracic surgeons) also pose significant challenges. Recognizing and detailing these additional hurdles provides a more comprehensive understanding of the intricacies involved in foreign body removal and underscores the importance of tailored approaches to optimize patient outcomes.

Historically, the journey of managing airway foreign bodies has witnessed several milestones. The pioneering effort by Gustav Killian stands out, as he marked the first successful extraction of an airway foreign body. Utilizing an esophagoscope, Killian retrieved a bone fragment from the right main bronchus of a 63-year-old male, laying the foundation for future bronchoscopic interventions [2]. 

Modern medicine offers a range of tools for this purpose, with rigid and flexible fiberoptic bronchoscopes being at the forefront. Both modalities, while effective, come with their unique set of advantages and challenges. Rigid bronchoscopy, traditionally the gold standard, provides a clear path for removal but requires general anesthesia. By contrast, the flexible fiberoptic bronchoscopy offers versatility, especially in navigating distal airways, and can be performed under conscious sedation. This study delves into our institutional experience with the latter, elucidating its efficacy and safety profile in adult foreign body retrievals.

## Materials and methods

We conducted an extensive retrospective analysis of bronchoscopic procedures performed in the Department of Pulmonary Medicine at the All India Institute of Medical Sciences, Jodhpur, India. The study was initiated after receiving full approval from the Institutional Ethics Review Committee (IEC no. 2019/1998), ensuring compliance with ethical standards and patient confidentiality protocols.

The study encompassed a detailed review of our medical records database, spanning from January 2018 to August 2019. This period was chosen based on the availability and completeness of the data. The primary aim was to collate comprehensive information regarding the utilization of bronchoscopy for suspected cases of foreign body aspiration.

We meticulously compiled data on the total number of bronchoscopic examinations conducted during the study period. Among these, particular attention was paid to instances suspected of foreign body aspiration. For each of these cases, we noted whether a foreign body was indeed visualized during the procedure. We detailed the exact anatomical location of the foreign body within the respiratory tract and described the techniques employed for its retrieval, including the use of forceps or suction devices as necessary.

Moreover, our study included thorough documentation of immediate adverse events occurring during or shortly after the procedure, as well as any complications noted up to six weeks post-retrieval. This approach provided a comprehensive overview of the safety and efficacy of the bronchoscopic interventions performed.

In conjunction with these data, we collected demographic information (age, gender, and residence) of the patients involved, along with their detailed clinical histories, which included symptoms of aspiration, duration of symptoms before bronchoscopy, and any previous attempts at removal. Radiological findings prior to the bronchoscopies were also reviewed, encompassing X-rays and CT scans, to assess the visibility and impact of the foreign bodies.

Data analysis was performed using statistical software, which facilitated a quantitative assessment of the frequencies and characteristics of bronchoscopy for foreign body retrieval. This analysis helped identify any significant patterns or trends in the incidence of foreign body aspiration, the success rate of retrieval, and the occurrence of complications, thereby contributing valuable insights into the effectiveness of bronchoscopic techniques in our clinical setting.

## Results

Between January 2018 and August 2019, a total of 510 bronchoscopies were performed. Of these, 39 (7.6%) were conducted due to suspected foreign body aspiration. In 11 (2.1%) of these patients, an airway foreign body was visualized during the bronchoscopy. The average age of these patients was 44.27 ± 17.3 years, with the majority being male (63.6%). No specific risk factors for foreign body aspiration were identified in any of the patients. The most common location for the foreign bodies was the right intermediate bronchus (46%), followed by the left main bronchus (18%) and the left lower lobe bronchus (18%). All foreign bodies were organic, with the exception of one plastic object (Table 1).

**Table 1:** Characteristics of patients with airway foreign body aspirations

Parameter		Values
Age (Mean ± SD) (in years)		44.27 ± 17.3
Males, n (%)		7 (63.6%)
Location of foreign body	Right intermediate bronchus	5 (46%)
	Right middle lobe bronchus	1 (9%)
	Right lower lobe bronchus	1 (9%)
	Left main bronchus	2 (18%)
	Left lower lobe bronchus	2 (18%)
Type of foreign body	Organic	10 (91%)
	Plastic	1 (9%)
Modality used	Flexible fibreoptic	8 (72.7%)
	Rigid	2 (18.2%)
	Surgery (Lobectomy)	1 (9.1%)

In eight patients (72.7%), the foreign body was successfully extracted using a flexible fibreoptic bronchoscopy. However, in two patients (18.2%), a rigid bronchoscope was required. One patient, who had a foreign body in the right lower lobe bronchus, had to undergo a right lower lobectomy due to the failure of both flexible and rigid bronchoscopy techniques. The foreign body in this patient was obscured by excessive granulation tissue. 

The flexible fibreoptic bronchoscopy (Olympus BF 1T150) was performed orally. For four patients, the foreign body retrieval was done in two stages because of the significant granulation tissue surrounding the foreign body. Electrocautery was applied during the initial phase to reduce the granulation tissue. These patients were administered dexamethasone (4 mg) three times daily for three days. In a subsequent bronchoscopy after three days, the foreign body was successfully retrieved in these patients. Various extraction tools were employed for the removal. A Fogarty catheter was utilized in five patients, grasping forceps in eight, and a Dormia basket in two. 

Using the flexible fibreoptic bronchoscopy for foreign body retrieval proved safe with minimal immediate complications. Mucosal bleeding was noted in three patients. For two of these patients, the bleeding ceased spontaneously, while the third required cold saline for control. Another patient experienced an exacerbation of underlying COPD, resulting in type II respiratory failure post-bronchoscopy. However, no delayed complications were reported among the eight patients who underwent the flexible fibreoptic bronchoscopy procedure. Of the two patients treated with rigid bronchoscopy, one suffered a tear in the left main bronchus as a complication. A subsequent bronchoscopy, performed four weeks later, revealed a web-like stenosis in the other patient (Table 2).

**Table 2:** Retrieval techniques and complications in patients with airway foreign body impaction.

Sr. No.	Age	Sex		Presentation	Type of foreign body	Site of foreign body	Technique of retrieval	Complications and management
1.	58	Male		Right lower zone non resolving pneumonia	Organic	Right intermediate bronchus	Flexible fibreoptic bronchoscopy + Fogarty catheter + Grasping forceps	None
2.	18	Female		Right lower lobe collapse	Organic	Right intermediate bronchus	Flexible fibreoptic bronchoscopy + Electrocautery + Fogarty catheter + Grasping forceps	Mild mucosal bleed – controlled spontaneously
3.	74	Male		Right lower zone recurrent pneumonia	Organic	Right intermediate bronchus	Flexible fibreoptic bronchoscopy via endotracheal tube + Dormia basket	Type II respiratory failure -required invasive mechanical ventilation for 48 hours
4.	48	Male		Right lower zone non resolving pneumonia	Organic	Right intermediate bronchus	Flexible fibreoptic bronchoscopy + Fogarty catheter + Grasping forceps	Mucosal bleed – controlled with cold saline
5.	66	Male		Dry cough	Organic	Right intermediate bronchus	Flexible fibreoptic bronchoscopy + Fogarty catheter + Grasping forceps	None
6.	48	Female		Right middle lobe broncheictasis	Organic	Right middle lobe bronchus	Flexible fibreoptic bronchoscopy + Fogarty catheter + Grasping forceps	None
7.	28	Female		Left lung collapse	Organic	Left main bronchus	Rigid bronchoscopy + Grasping forceps	Left main bronchus tear – managed conservatively
8.	55	Female		Left lung collapse	Organic	Left main bronchus	Rigid bronchoscopy + Grasping forceps	Web like stenosis of left main bronchus – treated with electrocautery and balloon dilatation
9.	37	Male		Left empyema	Organic	Left lower lobe bronchus	Flexible fibreoptic bronchoscopy + Grasping forceps	None
10.	31	Male		Dry cough	Inorganic (Plastic)	Posterior basal segment of left lower lobe bronchus	Flexible fibreoptic bronchoscopy + Electrocautery + Fogarty catheter	Mild mucosal bleed – controlled spontaneously
11	24	Male		Dry cough	Organic	Right lower lobe bronchus	Surgery due to presence of extensive granulation tissue	Nil

**Figure 1:**
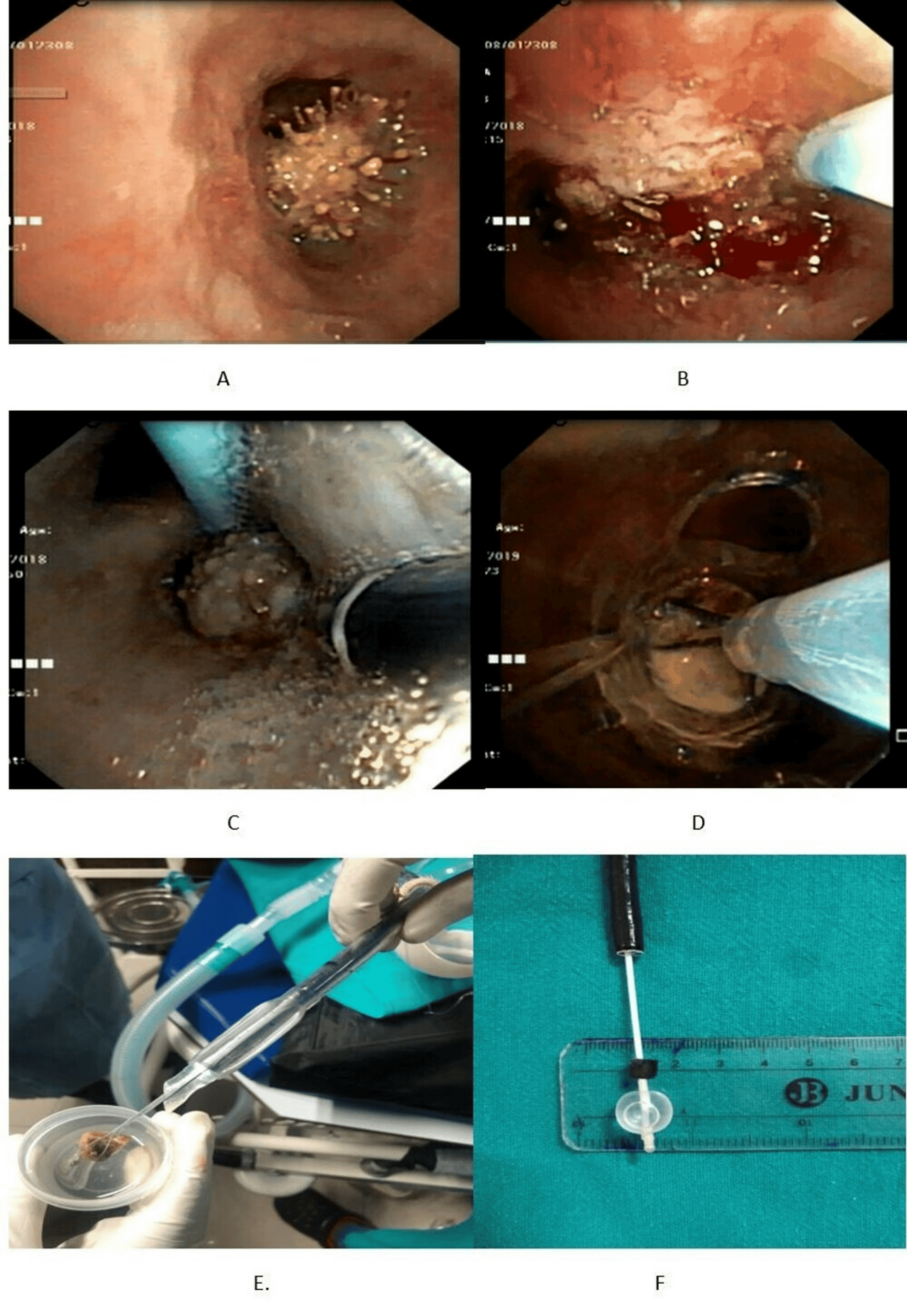
A) foreign body in the right intermediate bronchus in case 2. B) Cautery being used to reduce granulation tissue surrounding the foreign body in case 2. C) Foreign body being displaced into the main bronchus with forgarty and grasping forceps being used to grasp the foreign body in case 2. D) Foreign body trapped in a Dormia basket in case 3. E) Foreign body trapped in a Dormia basket removed enbloc along with a bronchoscope and endotracheal tube in case 3. F) Plastic foreign body mounted on a Fogarty catheter in case 10.

Figure 1 illustrates selected images of the retrieved foreign bodies and the extraction tools employed. In Figure 1A, a foreign body is visible in the right intermediate bronchus in case 2. This demonstrates the common occurrence of foreign bodies in the right bronchial tree, likely due to its more vertical orientation and larger diameter. In Figure 1B, electrocautery is used to reduce granulation tissue surrounding the foreign body, as in case 2. This highlights the need for adjunctive procedures to facilitate foreign body removal. Figure 1C shows the foreign body being displaced into the main bronchus using a Fogarty catheter, with grasping forceps being employed to secure the foreign body, again as in case 2. This emphasizes the use of multiple tools and techniques to successfully extract foreign bodies. In Figure 1D, the foreign body is trapped in a Dormia basket in case 3, and Figure 1E depicts the foreign body being removed en bloc along with the bronchoscope and endotracheal tube in the same case. Finally, Figure 1F presents a plastic foreign body mounted on a Fogarty catheter in case 10, illustrating the variety of foreign body types and the tools required for their removal.

## Discussion

The dilemma of foreign body aspiration transcends age groups, but it predominantly impacts children and the elderly [1]. Adults, although less commonly afflicted, are susceptible, particularly when specific risk factors are at play. These encompass a spectrum from physiological factors such as loss of teeth to neurological conditions and lifestyle choices including altered consciousness, alcoholism, sedative use, Parkinsonism, dementia, seizures, post-stroke consequences, CNS malignancies, amyotrophic lateral sclerosis with bulbar palsy, and mental retardation [2]. Intriguingly, our cohort lacked identifiable predisposing factors, emphasizing the unpredictable nature of such events. 

While children often manifest acute symptoms after aspiration, adults might adopt a sub-acute or even a chronic trajectory, which includes manifestations like non-resolving pneumonia, recurrent chest infections, or lobar collapse. Occasionally, the foreign body becomes a silent intruder, only to be incidentally discovered during unrelated clinical evaluations [3]. Such insidious cases underline the need for clinicians to remain vigilant, especially in the adult demographic. 

Foreign body categorization based on their nature (organic, inorganic, mineral, or miscellaneous) can influence their retrieval strategy. In our dataset, the vast majority (91%) were organic, with a single instance of a plastic foreign body. Anatomically, the right bronchial tree, owing to its vertical orientation and wider diameter, is the more likely lodging site. This is mirrored in our findings and corroborated by several other studies [4,5,6,7,8]. Notably, Sehgal et al.'s systematic review pinpointed the right lower lobe bronchus as the primary culprit, followed by the right intermediate bronchus [9]. 

Historically, the development and usage of bronchoscopes, both rigid and flexible, have evolved to enhance the precision and safety of foreign body retrieval. Rigid bronchoscopy, introduced by Gustav Killian, offers a more secure airway with potent suction capabilities, whereas the flexible bronchoscope, popularized for foreign body retrieval by Ikeda and subsequently Zvala et al. in animal models [10], presents as a more versatile tool especially suitable for outpatient settings. Each modality has its merits and drawbacks, as detailed in Table 3.

**Table 3:** Pros and cons of flexible and rigid bronchoscopy for use in patients with foreign body aspirations

Modality	Pros	Cons
Flexible bronchoscopy	Readily available	Does not provide a secure airway
	Easy to perform	Risk of foreign body loss during procedure
	Can be performed under conscious sedation	Risk of damage to the mucosa during removal of sharp foreign bodies
	Airways upto segmental bronchus can be visualised	
Rigid bronchoscopy	Provides secure airway	Not readily available
	No risk of damage by sharp ends of foreign body	Labour intensive
	Flexible bronchoscope can be passed through the barrel	Require additional skills and learning
		Requires general anaesthesia
		Only central airways can be visualised

A systematic review encapsulated the prowess of flexible bronchoscopy, citing a commendable success rate of 61-100% [9]. Our results, indicating a 72.7% success rate with flexible fibreoptic bronchoscopy, further solidify its standing. When faced with stubborn foreign bodies resistant to extraction by the flexible bronchoscope, the rigid counterpart or even surgical intervention may be necessary [3,7,9,11-14]. 

In terms of procedural nuances, oral entry is preferred during extraction via a flexible bronchoscope, facilitating en bloc removal. Diverse extraction tools, such as forceps, baskets, snares, cryoprobes, or balloon catheters, offer unique advantages depending on the nature and location of the foreign body. Baskets and cryoprobes are particularly useful for removing friable foreign bodies. The catheter, with its closed basket, is passed distal to the foreign body, and then opened to capture the foreign body before being removed. The cryoprobe tip is allowed to come into contact with the foreign body and is then frozen. This facilitates the en bloc extraction of the foreign body alongside the cryoprobe and bronchoscope [15,16]. Furthermore, granulation tissue surrounding the foreign body, as seen in four of our patients, necessitates additional considerations, such as steroid administration or electrocautery, for efficient retrieval. 

Post-extraction, an immediate re-evaluation of the airway is crucial. It serves multiple purposes: ensuring no residual foreign bodies, assessing post-procedural complications, and verifying the patency of distal airways. Our study highlights the importance of monitoring for complications, such as bleeding (observed in three of our patients), and the necessity of a follow-up airway examination, which in our cohort revealed complications like web-like stenosis.

It is essential to acknowledge certain limitations of our study. First, this study was confined to a single-center setting, potentially limiting the generalizability of our findings. Second, our study involved a relatively small number of patients, which may not capture the full spectrum of clinical scenarios and might reduce the statistical power to draw broader conclusions. The absence of a control group is another significant limitation, making it difficult to determine the baseline risk or to robustly assess the comparative effectiveness of the bronchoscopy techniques used. This prevents more definitive conclusions regarding the efficacy of the intervention compared to other methods or no interventions. The reliance on medical record reviews introduces variability in data recording and interpretation, possibly leading to biases that could affect the study's accuracy. Finally, we lacked certain advanced retrieval tools, most notably cryoprobes, that can facilitate the removal of challenging foreign bodies. Future research should aim to incorporate multiple centers, larger patient cohorts, and a broader range of endoscopic retrieval devices. Such studies would provide more definitive guidance on best practices and help further refine strategies for safe and effective airway foreign body extraction.

## Conclusions

Foreign body aspiration in adults requires a proactive, versatile, and evidence-based approach to diagnosis and management. Our study reinforces the effectiveness of flexible bronchoscopy as a primary intervention, with rigid bronchoscopy serving as an alternative when needed. The use of diverse extraction tools enhances procedural success. Future multicenter studies with larger cohorts are essential to establish best practices and optimize strategies for safe and effective management.
